# Molecular Characterization and Phylogenetic Analysis of Porcine Epidemic Diarrhea Viruses Associated with Outbreaks of Severe Diarrhea in Piglets in Jiangxi, China 2013

**DOI:** 10.1371/journal.pone.0120310

**Published:** 2015-03-19

**Authors:** Deping Song, Dongyan Huang, Qi Peng, Tao Huang, Yanjun Chen, Tiansheng Zhang, Xiaowei Nie, Houjun He, Ping Wang, Qinglan Liu, Yuxin Tang

**Affiliations:** 1 Department of Preventive Veterinary Medicine, College of Animal Science and Technology, Jiangxi Agricultural University, Nanchang, Jiangxi, China; 2 Jiangxi Academy of Agricultural Sciences, Nanchang, Jiangxi, China; Iowa State University, UNITED STATES

## Abstract

Porcine epidemic diarrhea (PED), caused by porcine epidemic diarrhea virus (PEDV), is a highly contagious, acute enteric viral disease of swine characterized by vomiting, watery diarrhea, dehydration and death. To identify and characterize the field PEDVs associated with the outbreaks of severe diarrhea in piglets in Jiangxi, 2013, the complete genome sequences of two representative strains of PEDV, designated CH/JX-1/2013 and CH/JX-2/2013, were determined and analyzed. The genome sequences of both emergent Jiangxi PEDV strains, CH/JX-1/2013 and CH/JX-2/2013, were 28,038 nucleotides in length excluding 3’ poly (A) tail. Compared to the PEDV CV777 strain, CH/JX-1/2013 and CH/JX-2/2013 had some unique genetic characteristics in the proximal region of the 5´-UTRs. Phylogenetic analysis of the complete genomes and the structural proteins revealed that CH/JX-1/2013 and CH/JX-2/2013 had a close relationship with post-2010 Chinese PEDV strains and US strains identified in 2013. The nucleotide identity between the two Jiangxi strains (CH/JX-1/2013 and CH/JX-2/2013) and 30 strains of PEDV identified ante-2010 and post-2010 ranged from 96.3–97.0% and 97.3–99.7%, respectively. Multiple nucleotide and deduced amino acid mutations were observed in the ORF1a/b, S, ORF3, E, M and N genes among the current field PEDV strains when compared to the CV777 strain. Some of the mutations altered the amino acid charge and hydrophilicity, and notably, there was an amino acid substitution in the middle of one neutralizing epitope (L1371I) of the S gene of both CH/JX-1/2013 and CH/JX-2/2013. Taken together, the accumulated genetic variations of the current field PEDV strains might have led to antigenic changes of the viruses, which might confer the less effectiveness or failure of the CV777-based vaccines currently being widely used in Jiangxi, China.

## Introduction

Porcine epidemic diarrhea (PED) is a highly contagious, acute enteric viral disease of swine characterized by vomiting, watery diarrhea, and dehydration and has become a devastating issue in many pig-raising countries [1]. PED was first reported in feeding and fattening pigs in England in 1971 [2] and then emerged in many European and Asian countries [3,4]. In late April 2013, PED was first confirmed in the United States, and quickly spread to all over the country [5–7]. In China, PED was identified as a sporadic viral enteric disease in pig-herds before 2010 [8–10]. However, outbreaks of PED with an increased severity of diarrhea, vomiting and dehydration have occurred in China since 2010, and the disease approached a mobility of as high as 100% and a mortality of 80–100% in piglets less than 10 days old, being recognized as a devastating illness causing death in neonatal piglets [11,12]. Pig farms in Jiangxi, one of the major pig-raising provinces located in eastern China, were severely affected by the outbreaks of PED accompanying with certain unique characteristics. Unlike its intrinsic seasonal epidemics ever seen before 2010, the PED prevalent in Jiangxi in 2013 has emerged all year round even in the midst of summer, and much worse, some of pig-raising farms repeatedly got affected from the disease, resulting in massive deaths of neonatal piglets. Likewise, the pigs vaccinated with inactivated CV777-based vaccines could also get affected with the disease [8,12]. The epidemiology of the 2013 PED outbreaks in Jiangxi suggested genetic/antigenic variations of PED virus (PEDV), the etiologic agent of PED, might occur so that the antigenicity, immunogenicity and virulence of the PEDVs had altered. Hence, in this study, we attempted to reveal the molecular characterizations of the PEDV strains currently circulating in Jiangxi, China and elucidate their phylogenetic relationship with PEDV vaccine strains and other reference strains.

PEDV is an enveloped, positive-sense, single-stranded RNA virus with large nested-set arrangement of the subgenomic mRNAs (sgmRNA), classified under the genus *Coronavirus* within the family *Coronaviridae*, order *Nidovirales* [13,14]. The genome of PEDV is approximately 28 kb nucleotides (nt) in length, containing 5´untranslated region (UTR), 3´UTR with a polyadenylated tail and at least seven open reading frames (ORFs), arranged in the order of 5´UTR-ORF1a/1b-spike glycoprotein (S)-hypothetical protein gene (ORF3)-envelope (E)-membrane (M)-nucleocapsid (N)-3´UTR [15,16]. The 5´UTR is about 291–296 nt, and the 3´UTR is about 334 nt in length. The polymerase gene, occupying about 5´two thirds of the complete genome, consists of ORF1a and ORF 1b, which encodes the polyprotein 1ab (PP 1ab). The four structural protein genes, occupying the 3´one-third part of the PEDV genome, encode S, E, M, and N proteins, respectively. The hypothetical accessory protein ORF3 is encoded by the gene located between S- and E-protein encoding regions. Previous analyses on the entire genome sequences indicated that the recent PEDV field strains circulating in China were genetically different from CV777 strain, from which vaccines have been generated and widely used in China for years [17–21].

In this study, a total of 125 fecal and intestinal samples from neonatal piglets with severe diarrhea from premises in different districts of Jiangxi Province, China were collected. Of which, 96 were detected to be positive for PEDV by a reverse transcription polymerase chain reaction (RT-PCR) established in our laboratory (S1 Table). To address the potential genetic/antigenic variations and phylogenetic characteristics of the PEDV strains associated with 2013 Jiangxi PED outbreaks, the complete genome of two representative intestinal specimens from 96 PEDV positive samples identified were sequenced and analyzed.

## Materials and Methods

### Ethics statement

The ethics committee of Jiangxi Agricultural University approved the animal protocol for this study (protocol number P-2013-03). All the procedures involving animals in this study were carried out in accordance with The Care and Use Guidelines of Experimental Animals established by the Ministry of Agriculture of China. Intestinal and fecal samples were collected according to the approved procedures. The intestinal samples used in this study were obtained from the dead piglets and the fecal samples were non-invasively collected immediately after excretion from healthy and diarrheal pigs from premises with PED outbreaks in Jiangxi, China (S1 Table).

### Virus identification

Fecal and intestinal samples (N = 125) were collected from < 10-day-old suckling piglets with severe watery diarrhea, vomiting and dehydration from different premises in Jiangxi Province, China (25–29°N and 114–118°E) in 2013. All the samples were examined by a RT-PCR established in our laboratory. Briefly, the total RNA was extracted from the samples using RNAplus Reagent (TaKaRa, Japan) following the manufacturer’s instructions. The one-step RT-PCR for determination of PEDV-positive samples was carried out using 50–200 ng of extracted RNA, forward primer (5´-GTATTGGTGGTGAGCGGAAT-3´), reverse primer (5´- CCTGTTCCGCCATTCTATCA-3´) and OneStep RT-PCR kit (Qiagen, Valencia, CA, USA) according to the manufacturer’s protocol. To eliminate possible co-infections caused by porcine transmissible gastroenteritis virus (TGEV), porcine rotavirus (PoRV), and other common pathogenic intestinal pathogens, differentiation assays were performed using standard protocols. Ninety-six out of 125 samples were tested positive for PEDV, and all samples had been confirmed to be free of TGEV, PoRV and other common enteric pathogens (data not shown). To address the genetic/antigenic variations, and phylogenetic characteristics of PEDV strains associated with 2013 Jiangxi PED outbreaks, two representative PEDV strains, designated CH/JX-1/2013 and CH/JX-2/2013 (the GenBank accession numbers are KF760557 and KJ526096, respectively), were used for sequencing the full-length genome.

### Full-length genome sequencing

Total RNAs were extracted from the feces and small intestinal homogenates by RNAplus Reagent (TaKaRa, Japan) according to the manufacturer’s instructions. The concentrations of the extracted RNAs were measured by NanoDrop 2000 spectrophotometer (Thermo Scientific, USA) and then stored at −80°C until use. The first-strand cDNA synthesis was performed at 42°C for 50 min and then 95°C for 5 min to inactivate the M-MLV reverse transcriptase (TaKaRa, Japan) and followed by 4°C for 5 min. The entire PEDV genome was amplified by 33 pairs of primer designed with Primer 3 software (http://primer3.ut.ee/) based on the conserved regions determined by a multiple alignment analysis of the reference strains and CV777 (S2 Table). Fragments were amplified on the conditions of a denaturation at 94°C for 4 min, 35 cycles (94°C x 45 sec, 53°C x 45 sec, 72°C x 1.5 min), and then with a final extension at 72°C for 10 min. PCR products obtained were subjected to gel purification using a gel extraction kit (TaKaRa, Japan), and afterwards cloned into pMD 18-T vectors (TaKaRa, Japan) following the manufacturer’s protocols. Five positive clones of each amplicon were submitted to a commercial sequencing company (Sangon Biotech, Shanghai, China) for sequencing at both directions by Sanger sequencing methodology. The 5’- and 3’- RACE for the determination of the terminal sequences of both CH/JX-1/2013 and CH/JX-2/2013 were performed by using 5’/3’ SMARTer RACE kit (Clontech, Beijing, China) following the manufacturer’s instructions.

### Sequence and phylogenetic analysis

The raw sequence fragments were imported to SeqMan in DNAStar Lasergene V 7.10 (DNAStar, Inc., Madison, WI) for assembly and annotation. Nucleotide and deduced amino acid (aa) sequences of both CH/JX-1/2013 and CH/JX-2/2013 and 30 reference PEDV sequences retrieved from GenBank were comparatively analyzed. A summary of the background information of PEDVs used in this study is shown in Table 1. The complete genome sequences of CH/JX-1/2013 and CH/JX-2/2013 were deposited into GenBank. Phylogenetic trees based on the entire genomes, and deduced aa sequences of S, ORF3, E, M, and N genes were constructed using the neighbor-joining method of MEGA 5.2.2 (http://www.megasoftware.net/) with a bootstrap of 1,000 replicate datasets. Primary sequences of 5´-proximal region of 5´-UTRs (nt 42 to 133) were pairwise compared between the two Jiangxi strains (CH/JX-1/2013 and CH/JX-2/2013) and the reference strains. Antigenicity and hydrophilicity analyses based on the aa sequence from 1 to 350 at N-terminal of the S proteins were carried out by Protean software of DNAStar Lasergene V7.10 (DNAStar, Inc., Madison, WI).

**Table 1:** Summary of the background information of CH/JX-1/2013, CH/JX-2/2013 and 33 reference strains used in this study.

Strains	Countries	Accession Numbers	Collection date	Strain	Country	Accession Numbers	Collection date
CV777	Belgium	AF353511	1978	Attenuated vaccine ^b^	China	KC189944	2012
CH/JX-1/2013	China	KF760557	2013	SD-M^b^	China	JX560761	2012
CH/JX-2/2013	China	KJ526096	2013	SM98	South Korea	GU937797	1998
AH2012	China	KC210145	2012	JS-HZ/2012	China	KC210147	2012
AJ1102	China	JX188454	2011	LC	China	JX489155	2011
Attenuated DR13^b^	South Korea	JQ023162	2003	Virulent DR13	South Korea	JQ023161	1999
BJ-2011-1	China	JN825712	2011	ZJCZ4	China	JX524137	2012
CH/FJND-3/2011	China	JQ282909	2011	Chinju99	South Korea	AY167585	1999
CH/FJZZ-9/2012	China	KC140102	2012	83P-5	Japan	AB548621	1983
CH/GD-01	China	JX261936	2012	Brl/87	France	Z25483	1987
CH/GDGZ/2012	China	KF384500	2012	LZC	China	EF185992	<2006^a^
CH/S	China	JN547228	1986	IA1	USA	KF468753	2013
CH/ZMDZ/Y11	China	KC196276	2011	USA/Indiana/17846/2013	USA	KF452323	2013
GD-1	China	JX647847	2011	ISU13-22038-IA-homogenate	USA	KF650373	2013
GD-A	China	JX112709	2012	ISU13-22038-IA-P9	USA	KF650375	2013
GD-B	China	JX088695	2012	Co/13	USA	KF272920	2013
JS2008	China	KC109141	2008	13-019349	USA	KF267450	2013
JS2008new	China	KC210146	2008				

^a^ The accurate isolation year of LZC is unknown, but it is estimated to be before 2006 according to the GenBank submission date.

^b^ cell adapted PEDV strains.

## Results

### Complete genome sequence comparison and phylogenetic analysis

The full-length genome of CH/JX-1/2013 and CH/JX-2/2013 was 28,038 nt in length excluding the 3´poly (A) tail. The genomic organization of CH/JX-1/2013 and CH/JX-2/2013 was similar to the reference PEDV strains. There was a ribosomal frameshift positioned 9 nt upstream of the slippery sequence 12610UUUAAAC12616 during the aa translation of ORF1 a/b. Thus, nucleotides encoding the PP 1ab were confirmed to be ‘293–12601 and 12601–20637’ in both CH/JX-1/2013 and CH/JX-2/2013. The full-length sequence of S, ORF3, E, M and N gene of both CH/JX-1/2013 and CH/JX-2/2013 was 4,161 nt, 675 nt, 231 nt, 681 nt and 1,326 nt, respectively. CH/JX-1/2013 and CH/JX-2/2013 shared 99.9% nucleotide identity with each other. A total of 27 nt differences were identified when compared to the whole genome of CH/JX-1/2013 and CH/JX-2/2013, and 14 out of 27 mutations led to aa changes. Of which, most were located in ORF1b and S gene (data not shown). The results from the genome-wide comparative analysis showed that the nucleotide identity between the Jiangxi PEDV strains and strains identified post-2010 ranged from 97.3–99.7%, while the nucleotide identity with those determined ante-2010 (CV777, attenuated vaccine_KC189944, SM98, JS2008, JS2008new and LZC) ranged from 96.3–97.0% (S3 Table). A total of 26 unique nucleotide substitutions were identified between two Jiangxi PEDV strains (CH/JX-1/2013 and CH/JX-2/2013) and 30 reference PEDV strains, which resulted in 14-aa changes, and most of them were located in ORF1a, ORF1b and S gene. Two of the nucleotide substitutions led to aa changes along with the charge variations (Table 2).

**Table 2:** Unique nucleotide/amino acid substitutions among the coding regions and UTRs between CH/JX-1/2013, CH/JX-2/2013 and the reference strains^a^.

Gene or ORF		nucleotide^b^ changes				amino acid^c^ changes		
	Position in genome^a^	Reference strains	CH/JX-1/2013	CH/JX-2/2013	Position in aa sequence^b^	Reference strains	CH/JX-1/2013	CH/JX-2/2013
**ORF1a (293–12646)**	1152	T	C	C				
	1196	A	G	G	aa 300	N	S	S
	1311	G	T	T				
	4124	C	T	T	aa 1276	A	V	V
	5298	C	T	T				
	8032	C	T	T	aa 2579	**H**	**Y**	**Y**
	8605	G	T	T				
	9057	T	C	C				
	9267	G	T	T				
	9991	C	T	T	aa 3232	**H**	**Y**	**Y**
**ORF1b (12811–20637)**	13511	T	C	C				
	16502	T	C	C				
	17462	C	A	A				
	19240	A	G	G	aa 2142	K	R	R
	20112	G	T	T	aa 2433	V	F	F
	20404	C	T	T	aa 2530	A	V	V
	20443	C	A	A	aa 2543	P	Q	Q
**S (20634–24794)**	21650	C	T	T	aa 337	A	V	V
	21955	C	T	T				
	21937	C	T	T				
	22402	T	G	G	aa 587	D	E	E
	22485	T	A	A	aa 615	F	Y	Y
	22893	C	T	T	aa 751	T	I	I
	24779	C	A	A	aa 1380	L	I	I
**N (26379–27704)**	26614	A	T	T				
	27638	G	T	T	aa 408	V	L	L

^a^ No nucleotide or amino acid changed in 5’UTR, 3’UTR and ORF3, E, and M genes

^b^ All nt mutations in the genome, including both nonsynonymous and silent mutations

^c^ Amino acids changes of replicase and structural protein genes at the position numbered in accordance with the aa sequence of individual protein

Bold letters indicate the amino acids with changed charge.

**Fig 1:**
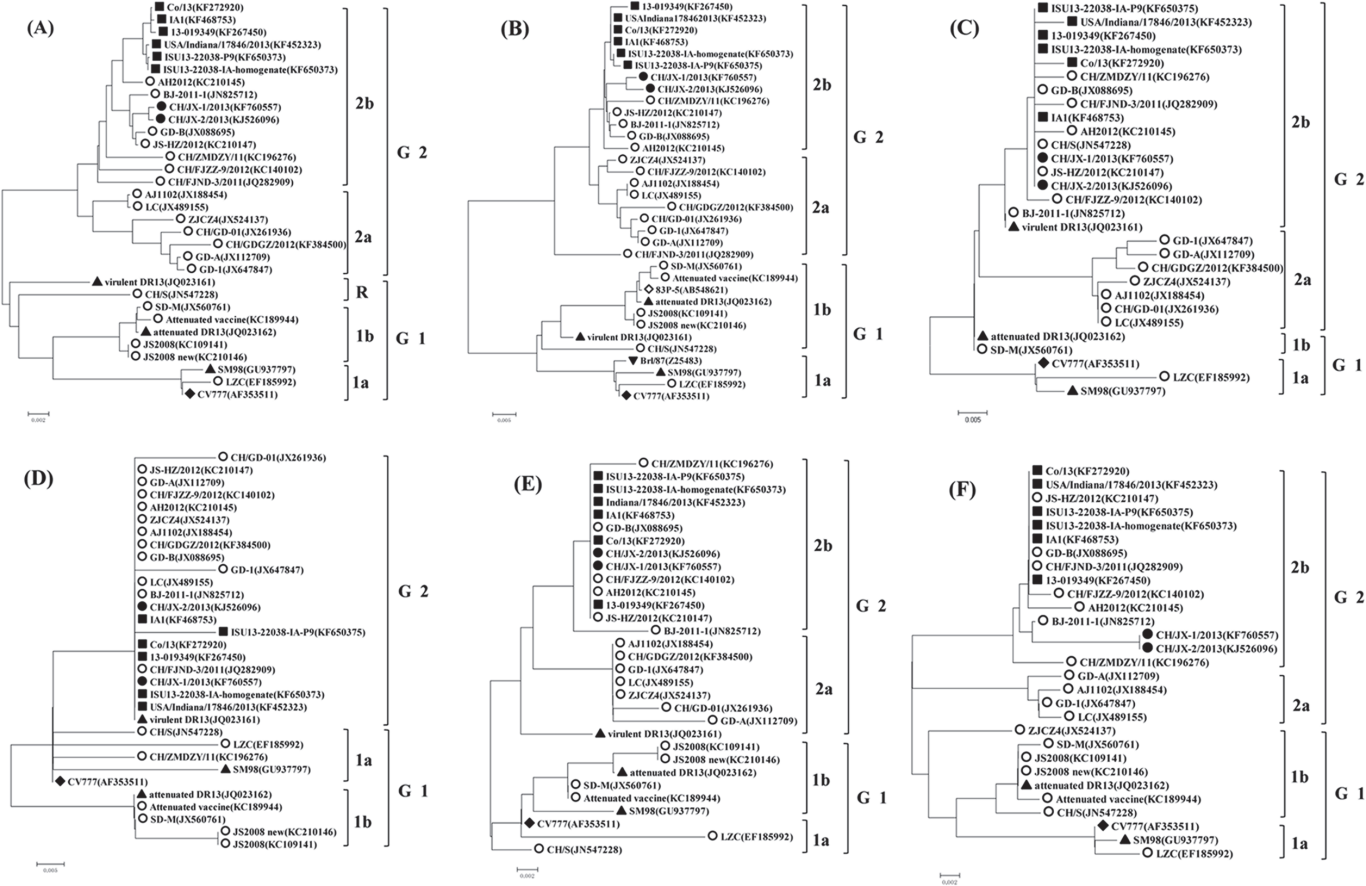
Phylogenetic trees based on the complete genome, aa sequences of structural proteins and ORF3 of PEDV strains. The trees were constructed by the distance-based neighbor-joining algorithm using MEGA 5.2.2 software. Bootstrap was set in 1,000 replicates with a value >70% to assess the significance of the tree topology. A bar of 0.002/0.005 indicates nucleotide or amino acid substitutions per site. “●” indicates the strains identified in this study, “○” indicates the strains from China, “◆” indicates the strains from Belgium, “■”indicates the strains from USA, “▲”indicates the strains from South Korea, “▼”indicates the strains from France, “◇”indicates the strains from Japan. 1A: Phylogenetic tree generated on the basis of nucleotide sequences of the complete genome of 33 PEDVs. 1B to 1F: Phylogenetic trees based on deduced amino acid sequences of S glycoprotein genes, ORF3, envelope, membrane, and nucleocapsid genes, respectively.

The phylogenetic tree based upon the full-length genome sequence of CH/JX-1/2013, CH/JX-2/2013 and 30 reference PEDVs indicated that the PEDV strains could be divided into two groups, designated for group1 (G1) and group 2 (G2): G1 could be further divided into three subgroups, i.e., 1a, 1b and R, a tentative cluster consisting of virulent DR13 isolated in South Korea in 1999 and CH/S isolated in China in 1986; and G2 was split into two subgroups, 2a and 2b (Fig. 1A). Notably, all the strains identified from 2011 to 2013 were fallen into G2; while the prototype of CV777, together with three Korean strains (SM98, virulent DR13 and attenuated DR13), and four earlier Chinese strains (JS2008, JS2008new, LZC and CH/S) were fallen into another group, i.e., group 1. Genetic characteristics were observed between the two groups: 1) compared to genome sequences of the members in G1, four insertions, 20803G, 20810CAGGGTGTCAA20820, 20830G, 21042AAT21044 and two deletions, 20842A, 21097CGTGAT21102, existed in the N-terminal domain (NTD) of the S protein in G 2 members; 2) the three field PEDV strains of JS2008, JS2008new and SD-M together with two attenuated PEDV strains, DR13 and vaccine_KC189944, were clustered into subgroup 1b. All of the five PEDV strains aforementioned had 24-nt deletions in nsp3 of ORF1a and 49-nt deletions in the C terminus of ORF3; 3) CH/JX-1/2013 and CH/JX-2/2013 along with three Chinese strains (GD-B, JS-HZ2012 and BJ-2011-1) were clustered into an independent clade, and CH/JX-1/2013 and CH/JX-2/2013 had 99.7%, 99.7% and 99.6% nucleotide identity with the three Chinese strains, respectively. These strains shared six additional unique nucleotide substitutions (T227A, C2342T, G2346T, T2724C, T6331C, C7233T) with the rest of reference PEDV strains. Of which, one led to an aa change (T682M in ORF1a, from hydrophilic polarity T to hydrophobic polarity M).

### Comparative analysis of structural genes

The full-length of S gene of CH/JX-1/2013 and CH/JX-2/2013 was 4,161 nt in size, which was 9-nt longer than that of the prototype of PEDV CV777 strain. The results from nucleotide sequence comparisons of the S gene of 34 strains of PEDV, including CH/JX-1/2013, CH/JX-2/2013 and other 32 reference PEDV strains from China, United States, UK, South Korea, Belgium, France and Japan showed that the two Jiangxi strains had a 99.8% nucleotide identity with each other, and 96.7% nucleotide identity with CV777. CH/JX-1/2013 and CH/JX-2/2013 shared 97.3–99.6% nt identity and 93.1–99.1% aa identity with the post-2010 PEDV strains, respectively. By contrast, the two Jiangxi strains showed only 93.5–95.0% nt identity, and 92.5–94.8% aa identity with the ante-2010 PEDV strains and attenuated vaccine strains, respectively (S4 Table).

**Fig 2:**
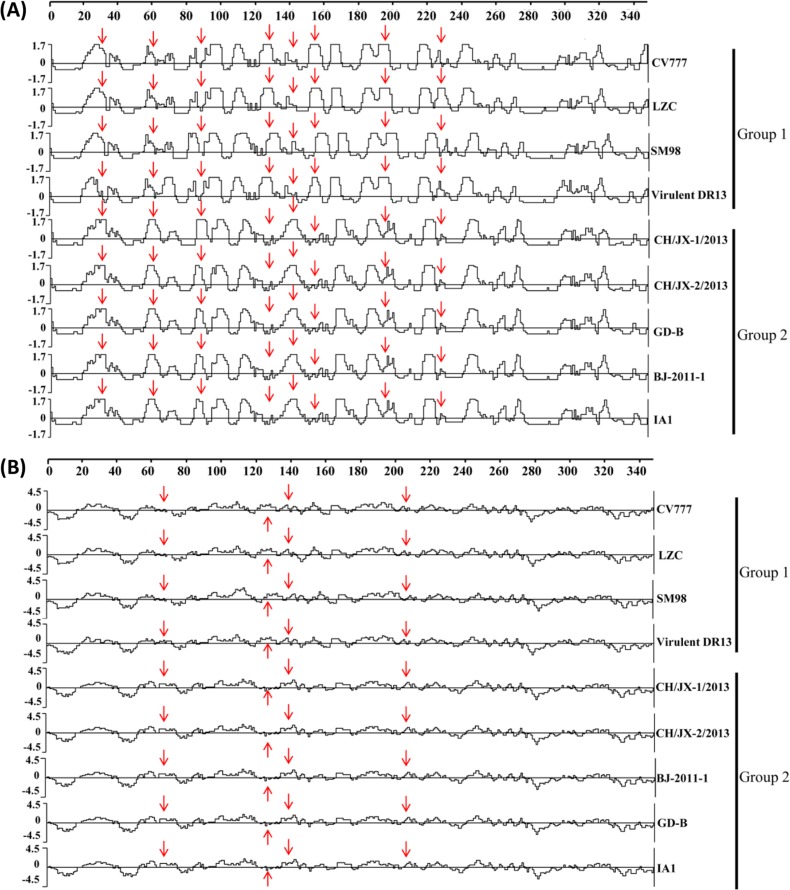
Antigenic and hydrophilic analyses of the amino acid sequences of partial S protein of nine PEDV strains. Antigenic index was calculated by using Protean of DNAStar Lasergene under the Jameson-Wolf method. Hydrophilicity plot was constructed by Kyte-Doolittle method. The arrows indicate the discrepancy of antigenic and hydrophilic plots of partial S protein of PEDV strains between phylogenetic group 1 (CV777, LZC, SM98 and virulent DR13) and group 2 (CH/JX-1/2013, CH/JX-2/2013, BJ-2011-1, GD-B and IA1) PEDV strains. Panel A: antigenic analysis, and panel B: hydrophilic analysis.

The phylogenetic tree based on the aa sequences of the S protein of 34 strains of PEDV showed that they could be classified into two major groups, and each group contained two subgroups (Fig. 1B). Both CH/JX-1/2013 and CH/JX-2/2013 belonged to subgroup 2b, which also included five newly identified US strains (13-019349, Co/13, IA1, ISU13-22038-IA-homogenate and ISU13-22038-IA-P9) and 13 Chinese strains identified at the same period or later than 2010. Compared to G1, there were two insertions (61VNST64 and 136N) and one deletion (155DG156) in the G2 members. Amino acid differences were also present between G1 and G2, and most of them were located in the area of aa 1 to 350 at N-terminal of the S protein (Table 3). The analysis based on aa from positioned 1 to 350 of the S protein suggested that the antigenicity and hydrophilicity of S protein of CH/JX-1/2013, CH/JX-2/2013 might have already changed due to the mutated amino acids with changes of charges and polarities (Fig. 2). Notably, an amino acid substitution was found in the middle of one neutralizing epitope (L1371I) of the S gene in both CH/JX-1/2013 and CH/JX-2/2013 when compared to CV777.

**Table 3:** Comparison of amino acid substitutions in S protein of the PEDV strains in phylogenetic group 1 and group 2.

Position in S protein	AA in group 1			AA in group 2		
	aa	charge	polarity	aa	charge	polarity
**55**	N	**M**	**−**	I	M	**−**
**65**	M	**M**	**−**	G	M	**+**
**57**	N	**M**	**−**	E	N	**+**
**58**	S	**M**	**+**	N	M	**−**
**59**	S	**M**	**+**	Q	M	**+**
**60**	S	**M**	**+**	G	M	**−**
**72**	E	**N**	**+**	P	M	**−**
**84**	D	**N**	**+**	R	P	**+**
**86**	K	**P**	**+**	S	M	**+**
**87**	R	**N**	**+**	G	M	**−**
**89**	Q	**M**	**+**	H^a^	P	**+**
**120**	I	**M**	**−**	T	M	**+**
**164**	I	**M**	**−**	S	M	**+**
**188**	H	**P**	**+**	Y	M	**−**
**201**	K	**P**	**+**	S	M	**+**
**202**	R	**P**	**+**	G	M	**−**
**203**	S	**M**	**+**	G	M	**−**
**228**	Y	**M**	**−**	S	M	**+**
**248**	S	**M**	**+**	P^b^	M	**−**
**554**	T	**M**	**+**	S	M	**+**
**599**	G	**M**	**−**	S	M	**+**
**964**	A	**M**	**−**	V	M	**−**
**1049** ^c^	S	**M**	**+**	A	M	**−**
**1178**	G	**M**	**−**	D^d^	N	**+**
**1237**	S	**M**	**+**	R	P	**+**

^a^ Strain CH/ZMDZY/11 is R at residue 89

^b^ Isolate CH/S is R at residue 248

^c^ Isolate CH/S is A at residue 1049, Strain CH/FJND-3/2011 is S at residue 1049

^d^ Strain CH/FJND-3/2011 is G at residue 1178.

“P” indicates positive charge

“N” indicates negative charge

“M” indicates neutral charge

“” indicates hydrophobic polarity

“+” indicates hydrophilic polarity

“ND” indicates the aa polarity is not determined yet.

The ORF3 of both CH/JX-1/2013 and CH/JX-2/2013 was 675 nt in length, encoding a protein of 224 aa. Both CH/JX-1/2013 and CH/JX-2/2013 shared 91.6–99.7% aa identity with other PEDV strains used in this study, and had the highest identity with a newly isolated US strain 13-019349 and a Chinese isolate JS-HZ/2012. A hexameric motif sequence of CTAGAC was observed in CH/JX-1/2013 and CH/JX-2/2013, positioning at 46 nt upstream of the initiator ATG in the ORF3 gene, which was similar to the situation of hexameric motifs XUA(A/G)AC existing in adjacent ORFs of PEDVs. Compared to CV777, a few nucleotide changes were found in both CH/JX-1/2013 and CH/JX-2/2013, and of which eight led to the aa changes (Table 4). The phylogenetic tree based on the deduced aa sequences of the ORF3 of the earlier European strains, CV777, Korean strains, US strains and Chinese strains identified ante or post-2010 revealed that those strains were grouped into two different groups (Fig. 1C). CV777 and two early strains SM98 and LZC, and two cell-adapted strains SD-M and attenuated DR13 were classified into group 1; while the 24 strains, including CH/JX-1/2013, CH/JX-2/2013, 15 Chinese PEDV strains, a Korean strain (virulent DR13), five newly determined US strains, and an early isolated Chinese strain CH/S were classified into group 2. Compared to CV777, all the other strains, except attenuated DR13, SM98, SD-M and LZC, shared 6-aa substitutions, at residues 21 (V21A), 54 (V54I), 79 (V79I), 92 (L92F), 101 (A101T, from hydrophobic polarity A to hydrophilic polarity T) and 166 (N166S, from hydrophobic N to hydrophilic S) in the ORF3 protein.

**Table 4:** Nucleotide and amino acid mutations in ORF 3 of CH/JX-1/2013 and CH/JX-2/2013 when compared to PEDV CV777.

	nucleotide change				amino acid change		
Position in ORF3 gene	CV777	CH/JX-1 /2013	CH/JX-2 /2013	Position in ORF3 protein	CV777	CH/JX-1 /2013	CH/JX-2 /2013
**54**	G	A	A				
**62**	T	C	C				
**63**	C	T	T	21	V	A	A
**160**	G	A	A				
**162**	T	C	C	54	V	L	L
**235**	G	A	A				
**237**	C	T	T	79	V	L	L
**238**	T	G	G	80	F	V	V
**243**	C	T	T				
**264**	C	T	T				
**274**	C	T	T	92	L	F	F
**301**	G	A	A	101	A	T	T
**369**	C	T	T				
**360**	T	C	C				
**393**	T	C	C				
**439**	C	T	T				
**450**	C	T	T				
**489**	C	T	T				
**537**	T	C	C	179	N	S	S
**546**	T	G	G	182	**H** ^a^	**Q**	**Q**

^a^ Bold letters indicate the amino acids with changed charge.

The E gene of CH/JX-1/2013 and CH/JX-2/2013 was 231 nt in size, encoding a protein of 76-aa. The sequence of E protein is extremely conserved. Although six nucleotide substitutions (T90C, C150T, TT165–166CC, A194G, and A198T) were observed in both CH/JX-1/2013 and CH/JX-2/2013 when compared to CV777, these mutations were all synonymous mutations. The phylogenetic tree constructed based on the deduced aa sequences of the selected reference PEDV strains showed that all the sequences could be classified into two groups, that is, Group 1 and Group 2 (Fig. 1D). The M gene of CH/JX-1/2013 and CH/JX-2/2013 had an ORF of 681 nt, encoding a 226-aa protein. Sequence comparison with 31 other PEDV strains revealed that the M protein was highly conserved, and CH/JX-1/2013 and CH/JX-2/2013 had a 97.8–100% aa identity with the reference strains of PEDV.

The phylogenetic analysis of M protein demonstrated that all PEDV strains were divided into two groups, i.e., Group 1 and Group 2 (Fig. 1E). Group 2 contained 15 Chinese strains, including CH/JX-1/2013 and CH/JX-2/2013, and six recent US strains. There was only one aa difference (V42A) in M protein between Group 1 and Group 2. Compared to CV777, CH/JX-1/2013 and CH/JX-2/2013 showed that three aa (E13Q, V42A and A214S) in M protein were changed. However, the aa substitution at position 13 (E13Q) altered the charge and polarity (from negative charge/hydrophilic polarity E to neutral charge/ hydrophobic polarity Q) of the N terminus of the M protein, which might have an impact on its antigenicity/immunogenicity.

The entire N gene of CH/JX-1/2013 and CH/JX-2/2013 was 1,326 nt long, encoding a protein of 441-aa. Sequence analyses revealed that the aa homology between CH/JX-1/2013 and CH/JX-2/2013 and other PEDV strains used in this study varied from 95–98.9%. The nucleotide and amino acid sequences of N protein were highly conserved and neither insertion nor deletion was found in all these PEDV strains analyzed in the study except a few sporadic mutations. Multi-alignment results of the aa sequences of the PEDV N proteins indicated that all the strains could be divided into two groups, i.e., Group 1 and Group 2. Each group contained two subgroups (Fig. 1F). The phylogenetic topology of N protein was similar to that of ORF3. Three specific aa changes were present between group 1 and group 2, at the residue 142 (A142T), 242 (H242L, from hydrophilic polarity I to hydrophobic polarity T) and 397 (Q397L). Five aa substitutions at the residue 84 (G84A), 205 (N205K, from neutral charge N to positive K), 381(L381P), 395 (L395Q, from hydrophobic L to hydrophilic Q), and 398 (H398N, from hydrophilic H to hydrophobic N) existed in subgroup 2a when compared with other three subgroups, i.e., subgroup 2b, subgroup1a and subgroup 1b.

### Nucleotide sequence comparison and structure prediction of 5´-RNA terminus

**Fig 3:**
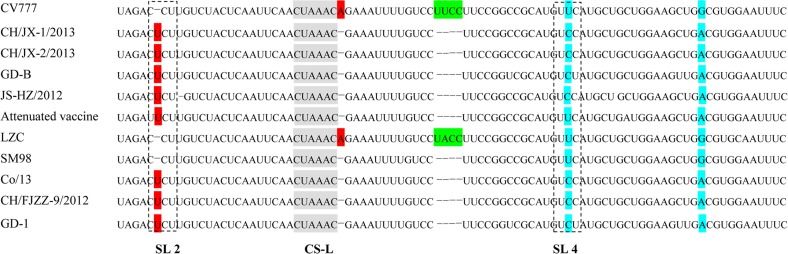
Comparison of primary sequence of 5´-proximal region (nt 42–133) in 5´UTRs between CH/JX-1/2013, CH/JX-2/2013 and reference strains. The core sequences (CUAAAC) of leader transcription-regulating sequences (CS-L) are highlighted in gray, nucleotide insertions are highlighted in red, and mutations are highlighted in blue. The stem-loop 2 (SL 2) and stem-loop 4 (SL 4) are marked with braces. An ‘A’ deletion is seen in both CH/JX-1/2013 and CH/JX-2/2013 identified in this study and other strains excluding CV777 and LZC.

The 5´UTRs of CH/JX-1/2013 and CH/JX-2/2013 were 292 nt in length, which was 4-nt shorter than that of CV777, resulting from 5-nt deletion and one nt insertion in the proximal region of 5´-UTRs of the two Jiangxi strains (Fig. 3). The core sequence (CUAAAC) of the PEDV leader transcription-regulating sequence (TRS) was extremely conserved with no nucleotide substitutions in all the PEDV strains. An ‘A’ deletion was observed immediately following the core sequence of these PEDV strains except the strains of CV777 and LZC. In comparison with CV777 and early isolated strains of SM98 and LZC, a ‘U’ insertion in loop 2 was found in both CH/JX-1/2013 and CH/JX-2/2013 and other six PEDV strains, such as GD-B, JS-HZ/2013, attenuated vaccine_KC189944, Co/13, CH/FJZZ-9/2012, and GD-1. The third nucleotide in loop 4 of recently determined strains of CH/JX-1/2013, CH/JX-2/2013, GD-B, JS-HZ/2012, Co/13, CH/FJZZ-9/2012 and GD-1 was a C, rather than a U at the same position in other four PEDV strains isolated earlier (CV777, Attenuated vaccine, LZC, and SM98). Compared to CV777, a 4-nt deletion (UUCC) existed in the stem region of SL4 of CH/JX-1/2013, CH/JX-2/2013 and other six PEDV strains. However this deletion would not impact the secondary structure of the SL4 as demonstrated by an analysis (data not shown). The 3’UTRs of both CH/JX-1/2013 and CH/JX-2/2013 were 334 nt in size, and no insertion and deletion was observed (Table 2).

## Discussion

PED affected massive pig farms in Jiangxi, China in 2013, causing substantial economic losses in the pig industry of Jiangxi. To elucidate the molecular characterization and phylogeny of the PEDV field strains associated with 2013 Jiangxi PED outbreaks, the entire genome sequences of two representative PEDV strains confirmed in Jiangxi were determined and analyzed. Phylogenetic analyses demonstrated that CH/JX-1/2013 and CH/JX-1/2013 defined in this study along with the strains determined post-2010 were clustered into group 2, whereas the strains detected ante-2010 were clustered into group 1. The results of phylogenetic analyses of those PEDV strains examined in this study were similar to that of described elsewhere [22]. CH/JX-1/2013 and CH/JX-1/2013 showed the highest nucleotide identity (>99%) with six newly confirmed US strains (IA1, Co/13, USA/Indiana, ISU13-22038-IA-homogenate, ISU13-22038-IA-P9, and 13-019349) and five Chinese strains (GD-B, AH2012, BJ-2011-1, CH/ZMD/ZY, and CH-HZ/2012) identified post-2010, suggesting these PEDV strains might have evolved from the same origin although the mechanisms of the evolution of the viruses are roughly unknown yet. Three field PEDV strains, JS2008, JS2008new and SD-M together with two attenuated PEDV strains, DR13 and vaccine_KC189944, were clustered into an independent cluster and showed 24-nt deletions in nsp 3 of ORF1a and 49-nt deletions in the C terminus of ORF3. These unique characteristics suggested that the three field PEDV strains might derive from the same source, and a recombination event might have occurred between these three viruses and the two vaccine strains in the same subgroup. Interestingly, a comparison analysis revealed that CH/JX-1/2013 and CH/JX-1/2013 had 99.7% nucleotide identity to the strain of GD-B (GenBank accession NO.JX088695) isolated from Guangdong, an adjacent province of Jiangxi, in which the severe PED outbreaks emerged in October 2010 [11,23]. Pigs are frequently traded between Jiangxi and Guangdong, which might be one of important factors causing the cross dissemination of PEDVs in this region. However, further study needs to be performed.

The findings from this study demonstrated that the 5´-UTR and ORF3 protein of CH/JX-1/2013 and CH/JX-2/2013 and all other PEDV strains analyzed were highly conserved, which were consistent with the studies reported previously [5,9]. In general, strict conservation of these regions is essential for the PEDV life cycle. It is known that the 5´-UTRs of coronaviruses form conserved RNA structural elements which are critical for viral replication, sgmRNA transcription, and translation [24]. Studies on the betacoronavirus have indicated that the stem-loop 2 (SL2) in 5´ UTR is extremely conserved in all coronaviruses, and plays a cis-acting reaction on transcription and replication, and more importantly the replication of the virus requires the conservative sequence and a firm number of nucleotides with specific properties in SL2 in 5´UTR [25]. Although the ‘U’ insertion in SL2 does not alter the RNA secondary structure, it might slightly affect the efficiency of virus replication, which needs to be addressed in the future studies. The core sequence (5´-CUAAAC-3´) in TRS of PEDV, which was also present in CH/JX-1/2013 and CH/JX-2/2013, has reported to be a determinant factor in transcriptional regulation in coronavirus because the synthesis of sgmRNA requires the appropriate tertiary structure of core sequence [26]. Both PEDV and TGEV belong to the *alphacoronavirus* genus within the *Coronaviridae* family and possess the conserved core sequence structure. Studies based on the infectious genomic TGEV cDNAs have proved that the nucleotides immediately flanking the TRS sequence could apparently affect the expression of the sgmRNA. And the nucleotides adjacent to core sequences of the leader TRS (CS-L) by the 3´region are more decisive for mRNA synthesis than nucleotides in the 5´ region. The motif of 5´-GAAA-3´ within 3´CS-L (5´-CUAAAC**GAAA**-3´) shows a higher expression than that of other motifs [27]. In respect of the PEDV, an ‘A’ deletion immediately followed the CS-L sequence in the 5´UTR of CH/JX-1/2013 and CH/JX-2/2013 as well as other post-2010 PEDV strains analyzed in the study and thus, this deletion formed a four base oligonucleotide (5´-GAAA-3) adjacent to the CS-L by the 3´region, which might up-regulate the sgmRNA expression in PEDV [24].

The S protein of PEDV is known to play pivotal roles in viral entry and inducing the neutralizing antibodies in natural hosts, and thus makes it become a primary target for the development of effective vaccines against PEDV [28–31]. It was demonstrated that there were significant genetic variations in the S gene between the newly determined PEDV field strains and early isolates [20,32]. The results in this study agreed with previous documentations. Additionally, CH/JX-1/2013 and CH/JX-2/2013 showed extremely high nucleotide and aa identity with each other but less to CV777 and attenuated strains (attenuated vaccine_KC18944 and attenuated DR13). There were significant aa differences between the G1 and G2 members, and most of them were located in NTD of the S protein of PEDVs. When compared to the group 1 strains of PEDV, CH/JX-1/2013 and CH/JX-2/2013, members of group 2, showed significant antigenic and hydrophobic differences, and some of which were located in the neutralizing epitope regions. Those genetic variations made the CH/JX-1/2013 and CH/JX-2/2013 and post-2010 field PEDV strains different from the ante-2010 strains, especially the CV777. It might explain why the recent PED outbreaks were significantly different from the previous sporadic outbreaks. Present PED became a devastating enteric viral disease causing substantial economic losses in the pig industry in major pig-raising countries in the world, and recently made it become a reportable disease by USDA [33]. Moreover, the present PEDV field strains displayed considerable genetic variations from CV777-based vaccine strain, a strain being widely used for production of PEDV vaccines in China for many years. Notably, Leucine, a highly hydrophobic aa residue in the middle of one neutralizing epitope (L1371I) of the S gene of CV777 was substituted by an Isoleucine, a higher hydrophobic index aa residue in both CH/JX-1/2013 and CH/JX-2/2013 strains. This variation might provide a possible mechanism for a poor protection on swine vaccinated with the CV777-based vaccines.

Park *et al*. [29] have reported that a reduced cell-culture-adapted PEDV strain (attenuated DR13, passage 100) has a truncated ORF3 of a 51-nt deletion, suggesting that this gene may be involved in cell tropism and is essential for the virulence of PEDVs. By contrast, CH/JX-1/2013 and CH/JX-2/2013, together with the post-2010 variant PEDV strains had an intact ORF3 of 675 nucleotides encoding a protein of 224 aa. The phylogenic analysis demonstrated that the ORF3-based tree had a similar topology with the one generated from the entire genome sequences. And thus the ORF3 of PEDV may serve as a useful target gene for phylogenetic analysis of newly emerging field PEDV strains since its small size. As recognized previously [34,35], there is a large deletion region existing in PEDV isolates attenuated DR13 (51-nt at the position of 245–295), SM98 (210 nt at the position of 1–210) and SD-M (51-nt at the position of 245–295), a Chinese strain propagated only four passages on cell lines.

In summary, the findings obtained in this study provide some insight into the genetic/phylogenetic variations and molecular characterizations of the Jiangxi field PEDV strains associated with the outbreaks in piglets in Jiangxi, China 2013. The comparisons of the full-length genome and the structural protein genes and deduced aa sequences revealed that two Jiangxi strains CH/JX-1/2013 and CH/JX-2/2013 defined in this study had a close relationship with the recent prevailing field PEDV strains in China and the United States. Differences at the level of the nucleotides and deduced aa between the present field PEDV strains and CV777, especially the aa substitutions in the neutralizing epitope of PEDV field strains, might have conferred the less effectiveness of the vaccines currently widely being used in China. It might be urgently needed to develop improved efficacious and safe vaccines against field PEDVs being currently circulating in China.

## Supporting Information

S1 Table Background information of fecal and intestinal samples collected from diarrheal/dead piglets from premises with PED in Jiangxi, China 2013. (XLS)

S2 Table Primers used for the amplification of full-length genomes of the PEDV strains CH/JX-1/2013 and CH/JX-2/2013. (XLS)

S3 Table Percent nucleotide identity for the complete genomes of 32 strains of PEDV. (XLS)

S4 Table Percent nucleotide and amino acid identities for the complete S genes of 34 strains of PEDV. (XLS)
